# Joint Unsupervised Learning of Depth, Pose, Ground Normal Vector and Ground Segmentation by a Monocular Camera Sensor

**DOI:** 10.3390/s20133737

**Published:** 2020-07-03

**Authors:** Lu Xiong, Yongkun Wen, Yuyao Huang, Junqiao Zhao, Wei Tian

**Affiliations:** Institute of Intelligent Vehicles, School of Automotive Studies, Tongji University, Shanghai 201804, China; xiong_lu@tongji.edu.cn (L.X.); 1933550@tongji.edu.cn (Y.W.); huangyuyao@tongji.edu.cn (Y.H.); zhaojunqiao@tongji.edu.cn (J.Z.)

**Keywords:** unsupervised learning, scene depth, ego-motion, ground segmentation, ground normal vector

## Abstract

We propose a completely unsupervised approach to simultaneously estimate scene depth, ego-pose, ground segmentation and ground normal vector from only monocular RGB video sequences. In our approach, estimation for different scene structures can mutually benefit each other by the joint optimization. Specifically, we use the mutual information loss to pre-train the ground segmentation network and before adding the corresponding self-learning label obtained by a geometric method. By using the static nature of the ground and its normal vector, the scene depth and ego-motion can be efficiently learned by the self-supervised learning procedure. Extensive experimental results on both Cityscapes and KITTI benchmark demonstrate the significant improvement on the estimation accuracy for both scene depth and ego-pose by our approach. We also achieve an average error of about 3${}^{\circ }$ for estimated ground normal vectors. By deploying our proposed geometric constraints, the IOU accuracy of unsupervised ground segmentation is increased by 35% on the Cityscapes dataset.

## 1. Introduction

Estimation of scene depth, ground plane and ground normal vector by camera sensors has been playing a key role in the field of automated driving and robotics. Together with the inter-frame motion estimation, they can provide a priori knowledge about a scene structure. Since such information is essential to tasks such as tracking [1], 3D object detection [2,3] and camera pose estimation [4], numerous approaches related to scene structure prediction and analysis have been proposed in recent years.

Generally, scene structures can be estimated either by the traditional geometric vision methods or by deep learning based approaches. For scene depth and ego-motion estimation, traditional methods typically leverage extensive feature matching in accordance with multi-view geometry while the results strongly rely on the matching accuracy and measured camera parameters [5,6,7,8]. In deep learning based approaches, especially in unsupervised learning, the input data is only continuous RGB video streams, and no additional supervised signals or labels are required. The inter-frame motion and scene depth can be estimated by the network itself through reconstructing the image from one frame to another w.r.t. photometric errors. In spite of impressive results achieved [9,10,11,12,13], one persisting problem for such methods is that dynamic objects do not conform to the original static scene assumption. In terms of ground structure estimation, traditional methods mainly leverage multi-view geometry, e.g., by fitting a large plane to the obtained 3D point cloud [14], or by estimating vanishing points and horizons using parallel lines on the ground [7]. These methods either require guidance by additional signals such as lidar points or strongly rely on intermediate scene element predictions, which are inherently less robust and difficult to apply to a wider range of scenarios. In deep learning methods, ground plane predictions are typically learned in a supervised fashion [15], yet the required supervision signal is usually cumbersome in data collection. In addition, the link between scene structures has been taken into consideration in recent researches [16,17], which is beneficial for the entire scene understanding. Given these circumstances, we propose a unified and completely unsupervised learning framework, which can estimate the scene depth, ego-motion, ground normal vector and ground segmentation simultaneously. This framework only needs continuous video streams and, thus, no annotation for the scene is required. To make full use of the information of different scene structures, we propose a joint learning process. In this process, the estimated depth is used to restore 3D coordinates for the corresponding image points. The ground segmentation network is trained using point labels refined by estimated ground plane and its normal vector. The corresponding loss is called ground self-learning loss. To make use of the ground information in dynamic scenes (with ego-motion), we propose the plane photometric loss, which considers plane transform errors between frames by the homography matrix. We also propose another loss to punish depth abnormality in regions near the vanishing point obtained by the ground normal vector. By filtering out points with abnormal depth values, the network model can be further optimized. We validate our approach on both Cityscapes [18] and KITTI [19] benchmark and a significant improvement on estimation accuracy for both scene depth and ego-pose has been proven. In comparison with other unsupervised methods, we achieve an accuracy gain of about $1.1^{\circ }$ in ground normal vector estimation and 35% in unsupervised ground segmentation, which further demonstrate the efficacy of our approach.

In this paper, our contributions are summarized as follows:

- An unsupervised learning framework is proposed, which can estimate the scene depth, ego-motion, ground normal vector and ground segmentation simultaneously.
- A joint learning process is proposed, which uses heterogeneous loss functions to boost the mutual information flow between the estimation of different scene structures.
- Extensive comparison experiments and ablation studies on public datasets are conducted and demonstrate the improvement of proposed approach on estimation of scene structures such as the depth, ego-pose, ground segmentation and ground normal vector.

## 2. Related Work

In accordance with the focus of this paper, related works are reviewed in following three aspects: depth and ego-motion estimation, unsupervised semantic segmentation, and ground normal vector estimation.

### 2.1. Depth and Ego-Motion Estimation

Traditional depth estimation methods mainly rely on multi-view geometry to perform 3D restoration through inter-frame registration [7]. With the development of deep learning, Convolutional Neural Networks (CNNs) are used for depth prediction. Eigen et al. [20] proposed a supervised approach with two networks to estimate depth in a coarse-to-fine order: one network makes global prediction of the entire graph, while the other refines the local information. Similar works can be seen in [21,22,23,24]. Although such methods work well, the cost of depth labels restricts these methods. To bypass this restriction, stereo image pairs are used in other works, where the left frame is reconstructed through the predicted depth and the known inter-frame pose, and the photometric error is used as the supervision signal during training [25,26]. Despite no explicit supervision, these methods require more accurate sensor calibration. Zhou et al. [9] first proposed an unsupervised framework only requiring video streams. In their approach, images are reconstructed using depth and inter-frame motion, which is estimated by a self-motion estimation network. However, this method is based on the assumption of static scene, where dynamic objects can contaminate the network prediction. In order to deal with dynamic objects, optical flow [12] and dynamic object segmentation [13] are added by researchers. These tricks improve the accuracy, but also increase the computation cost by employing many intermediate processes. Bian et al. [27] proposed the geometry consistency loss to reduce the relative depth error of the same spatial point in two frames. In their approach, the depth consistency is also used as the weight of the photometric error, reducing the impact of dynamic objects.

### 2.2. Unsupervised Semantic Segmentation

Unsupervised semantic segmentation can theoretically use the method of grouping. Typical approaches for grouping are information maximizing self-augmented training (IMSAT) [28] by maximizing the mutual information between data and its representation, and Deep InfoMax (DIM) [29], which maximizes the information between spatially preserved features and compact features. Deep clustering is also used in a lot of grouping approaches for image, and learns highly transferable intermediate features using overclustering as a proxy task. Yang et al. [30] iteratively learn convolutional network features and clusters with a recurrent framework. Their model offers promising performance on small datasets. However, these methods either introduce additional grouping criteria which increase the method complexity or are only proven effective on small datasets. In contrast, the unsupervised method proposed by Ji et al. [31] use mutual information loss for supervision and because the training process is not easy to degrade, it improves the ability on large datasets. In the approach of Ji et al. [31], they perform a random transform on a single image and send both the transformed image and the original one into the network respectively. After network processing, an inverse transform is performed on the transformed image. By maximizing the mutual information of corresponding pixel in both images, their model can better learn shallow semantic information.

### 2.3. Ground Normal Vector Estimation

Multi-view geometry is broadly used in traditional methods to estimate the structural feature of the plane in monocular images [32], e.g., by detecting the vanishing point and the horizon through parallel lines on the plane. However, this type of method heavily relies on the performance of manual features and is less robust. Other methods attempt to learn ground normal vectors for each pixel and cluster the results to obtain planes [16,17,33,34]. There are also indirect methods for obtaining normal vectors through 3D reconstruction [32,35,36]. But these methods are either for indoor scenarios or require surface labels. In a recent work, Man et al. [15] estimate the ground normal and depth in two separate streams. A consistency loss is added to enhance the accuracy of the normal vector. Although this work obtains better accuracy, it still requires labels for depth, normal vector and ground segmentation.

Our approach is based on preliminary works [27,31]. To enhance the ground segmentation performance, we add geometric priors (the ground normal) in the joint training process. Additionally, we add information of static ground plane and through the plane photometric loss we improve the performance of depth and pose estimation. The ground normal vector is naturally calculated by ground separation and its depth. Under the mutual promotion between subtasks, the estimation of depth, pose, ground normal vector and ground segmentation can be improved.

## 3. Proposed Method

**Figure 1:**
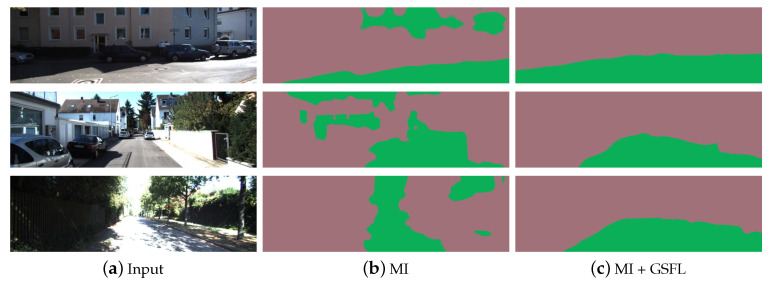
From left to right are: the input image, the ground segmentation result obtained only using the mutual information loss (MI), and the result by joint learning which uses the ground self-learning loss (GSFL). It can be seen that after adopting the geometric prior, the ground segmentation results have been enhanced.

Mutual information loss (MI) has been used for unsupervised segmentation in preliminary work [31]. However, for complicated traffic scenes, the result becomes unreliable, as shown in the middle of Figure 1. Because the supervision signal is insufficient, it can only learn shallow semantic features and give a very rough hypothesis about the ground. In our approach, we merge the ground segmentation with a structure from motion (SfM) framework, so that the tasks of the estimation of depth, ego-motion, ground segmentation and ground normal vector can promote each other. For this purpose, we add the ground self-learning loss $L_{r}$, the plane photometric loss $L_{H}$, and the depth abnormal punishment loss $L_{v}$. We also use the ground normal vector to improve the origin loss in the SfM framework.

**Figure 2:**
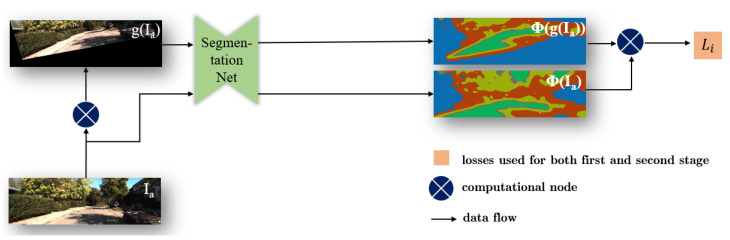
The original image $I_{a}$ undergoes a random transform to generate $g(I_{a})$. Both of them are then passed separately into the segmentation net, and yield the probability feature map $\Phi (g\left(I_{a}\right))$ and $\Phi (I_{a})$ to calculate the mutual information loss $L_{i}$.

**Figure 3:**
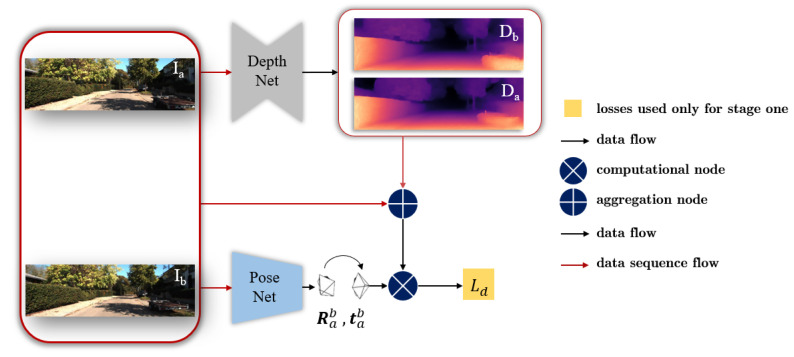
The unsupervised depth learning framework in our approach. Detailed description about its structure can be seen in the text.

**Figure 4:**
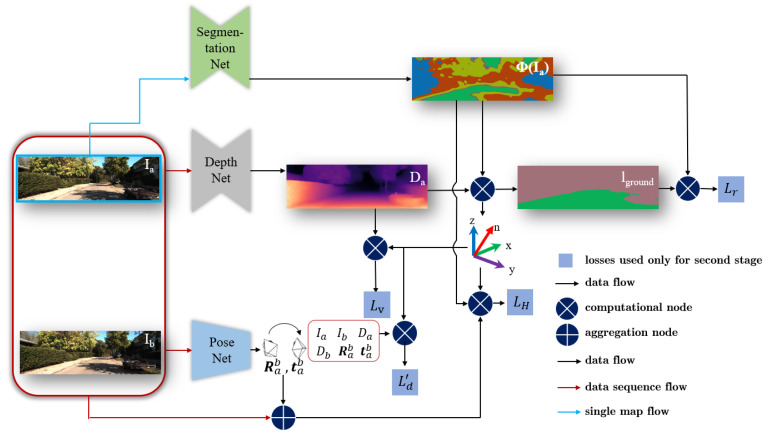
New losses $L_{r}$, $L_{H}$ and $L_{v}$ are added in the second learning stage. In comparison with the first learning stage, the normal vector is fused to improve the calculation of loss $L_{d}$, forming the new loss $L_{d}’$. Details about this learning stage can be seen in the text.

The training of our approach consists of two stages. The first stage is for pre-training the ground segmentation network (Figure 2) and the SfM framework (Figure 3), respectively. In the second stage, we perform the joint learning (Figure 4) with the aforementioned three losses and use the normal vector to improve the origin loss in SfM framework. Thus, our entire loss function is defined as

(1) $$L=L_{i}+L_{d}+L_{H}+L_{r}+L_{v},$$

where $L_{i}$ and $L_{d}$ respectively denote the mutual information loss and the scene reconstruction loss from the first training phase. In the second phase, the $L_{d}$ is improved to $L_{d}^{{}^{\prime }}$ which will be explained in the Joint learning part, and $L_{H},L_{r},L_{v}$ are adopted. Note that for an intuitive expression, the weight for each loss is implicitly integrated in their formulations (introduced in following subsections). By the above training procedure, the SfM framework can guide the learning of the ground segmentation. Through the ground self-learning loss and the depth abnormal punishment loss, the learned ground-related information feeds back to the SfM framework, and finally obtains a stronger scene structure estimation.

In following parts, the calculation of corresponding losses as well as training details are introduced. Specifically, the loss $L_{i}$ and $L_{d}$ used in the first learning stage will be introduced in Section 3.1 and Section 3.2, respectively. In Section 3.3, the loss $L_{r}$, $L_{H}$, $L_{v}$ will be represented, which are added in the second stage, and the improved loss $L_{d}^{\prime }$ will be described. Finally, in Section 3.4, a small summary of the entire framework will be expressed.

### 3.1. Self-Supervised Ground Segmentation

Here we adopt the mutual information loss to make a coarse prediction for the ground. By maximizing the mutual information loss of image pairs, image regions within the same semantic class should have similar probability distribution, and thus belong to the same semantic segment.

Given two discrete probability distributions **z** and $z^{\prime }$, the joint probability distribution matrix **P** can be calculated by function *h* as

(2) $$P=h\left(z,z^{\prime }\right)=\frac{zz^{\prime ⊤}+z^{\prime }z^{⊤}}{2}.$$

Thus, the mutual information [31] is expressed as

(3) $$MI\left(P\right)=\sum_{c=1}^{C}\sum_{c^{\prime }=1}^{C}P_{cc^{\prime }}\ln\frac{P_{cc^{\prime }}}{P_{c}P_{c^{\prime }}},$$

where $P_{c}$ represents the marginal probability of class *c* in **z** and $P_{c^{\prime }}$ represents the marginal probability of class $c^{\prime }$ in $z^{\prime }$. $P_{cc^{\prime }}$ denotes the joint probability for *c* and $c^{\prime }$. To extend this concept, the joint probability distribution matrix $P_{I_{a}}$ for the image $I_{a}$ and its transformed version $g(I_{a})$ (see Figure 2) can be defined as

(4) $$P_{I_{a}}=\frac{1}{|V_{i}|}\sum_{\left(x,y\right)\in V_{i}}h\left(\Phi {\left(I_{a}\right)}_{xy},\;g^{-1}{\left(\Phi \left(g\left(I_{a}\right)\right)\right)}_{xy}\right),$$

where g(·) represents a random set of image transform, including translation, rotation, hue change, etc. And $g^{-1}\left(\cdot \right)$ denotes the inverse transform. Term Φ(·) indicates the probability map generated by a segmentation network. Subscript xy denotes the image coordinate of point (x,y). Set $V_{i}$ contains valid points which remain in the image after the transform g(·) or $g^{-1}\left(\cdot \right)$. And $|V_{i}|$ denotes the number of points in this set.

For semantic segmentation, the mutual information loss $L_{i}$ can be expressed as

(5) $$L_{i}=-MI\left(P_{I_{a}}\right).$$

This method can only yield a very rough cluster as shown in the middle of Figure 1, which is far from enough in complex scenes. Thus, in our approach, this coarse ground segmentation is combined with the depth information to expand the ground part, i.e., to optimize the ground segmentation result by compensating the geometric information. The depth information is estimated by a SfM framework introduced below.

### 3.2. SfM Framework

The overall SfM framework in our approach is illustrated in Figure 3. The depth $D_{a}$ of frame $I_{a}$ is estimated through the DepthNet. Two consecutive frames $I_{a},I_{b}$ are sent to the PoseNet to estimate the ego-motion $\left[R_{a}^{b},t_{a}^{b}\right]$. Then, the depth $D_{a}$, pose $\left[R_{a}^{b},t_{a}^{b}\right]$ and frame $I_{b}$ are used to reconstruct frame $I_{a^{\prime }}$ to calculate the photometric loss. The smooth loss and geometry consistency loss are also added in the training. Therefore, the scene reconstruction loss function is defined as

(6) $$L_{d}=\alpha L_{p}^{\prime }+\beta L_{s}+\gamma L_{GC},$$

where $L_{p}^{\prime }$ is the depth constrained photometric loss, $L_{s}$ is the smooth loss and $L_{GC}$ is the geometric consistency. And α, β, γ are hyper-parameters for trade-off between individual losses.

The loss based on Structural Similarity (SSIM) [37] comprehensively considers the difference of lighting, contrast, and image structure, while the simple $L_{1}$ distance is more sensitive to illumination. The superiority of SSIM in image reconstruction has been proven in Bian et al. [27]. Thus, the loss based on SSIM is more suitable to be integrated in the photometric loss function. (Details can be seen in Appendix C.) So, $L_{p}$ is calculated as

(7) $$L_{p}=\frac{1}{|V_{d}|}\sum_{p\in V_{d}}\left(\;\left(1-\lambda_{s}\right){∥I_{a}\left(p\right)-I_{a^{\prime }}\left(p\right)∥}_{1}+\lambda_{s}\frac{1-{\mathrm{SSIM}}_{aa^{\prime }}\left(p\right)}{2}\right).$$

Here $|V_{d}|$ represents the number of successfully transformed image point *p*, $I_{a^{\prime }}$ is the reconstructed image (by obtained pose $\left[R_{a}^{b},t_{a}^{b}\right]$, next frame $I_{b}$ and depth $D_{b}$ in Figure 3) of image $I_{a}$. The loss based on SSIM describes the structural similarity between image $I_{a^{\prime }}$ and $I_{a}$. $\lambda_{s}$ is a hyper-parameter.

The smooth loss item $L_{s}$ is used to punish discontinuity of depth in local region and formulated by

(8) $$L_{s}=\sum_{p}{\left(e^{-\nabla I(p)}\cdot \nabla D\left(p\right)\right)}^{2}$$

with ∇ as a gradient operation.

The geometric consistency constraint $L_{GC}$ [27] is added to get better scale consistency. It is calculated by following equation:

(9) $$D_{\mathrm{diff}}\left(p\right)=\frac{\left|D_{a^{\prime }}\left(p\right)-D_{a}\left(p\right)\right|}{D_{a^{\prime }}\left(p\right)+D_{a}\left(p\right)},$$

(10) $$L_{GC}=\frac{1}{|V_{d}|}\sum_{p\in V_{d}}D_{\mathrm{diff}}\left(p\right).$$

$D_{a^{\prime }}$ is the reconstructed depth of $I_{a}$ and obtained by transforming $D_{b}$ (the depth of next frame $I_{b}$) according to the pose [$R_{b}^{a}$, $t_{b}^{a}$] and itself. This consistency constraint is used to optimize the original photometric loss $L_{p}$ by

(11) $$L_{p}^{\prime }=\frac{1}{|V_{d}|}\sum_{p\in V}\left(\left(1-D_{\mathrm{diff}}\right)\cdot L_{p}\left(p\right)\right).$$

### 3.3. Joint Learning

In this part, the learning process to merge ground estimation with the SfM framework by those three losses $L_{r}$, $L_{H}$ and $L_{v}$ will be introduced in details. The whole joint learning procedure is shown in Figure 4.

With the rough ground segmentation $\Phi (I_{a})$ and estimated depth $D_{a}$, the normal vector *n* of the ground plane is calculated by the Random Sample Consensus (RANSAC) method. A self-learning ground label $l_{ground}$ is obtained from points within a valid distance to the plane. $I_{ground}$ and $\Phi (I_{a})$ are used to calculate the cross-entropy loss $L_{r}$. By using this loss, the road surface segmentation is re-learned according to the geometry information. The normal vector *n* is used to calculate the vanishing point and an ROI. Abnormal depth values in this ROI are punished through $L_{v}$ and also eliminated in the calculation of $L_{d}^{\prime }$. At the end, the plane photometric loss $L_{H}$ is calculated with normal vector *n*, ground segmentation $\Phi (I_{a})$, successive frames $I_{a},I_{b}$, and pose estimation $\left[R_{a}^{b},t_{a}^{b}\right]$. This loss is used to optimize the pose model. More details about this learning procedure are described in following parts.

#### 3.3.1. Ground Self-Learning Loss

The depth itself actually contains a lot of structural information, especially for the ground. Using this loss, the coarse ground segmentation is combined with the depth information to refine the self-learning labels for ground segmentation network so that a better segmentation result can be restored.

Here the output of the ground segmentation network is processed by the softmax layer to obtain the probability map $\Phi (I_{a})$. Here we only consider a binary classification problem (i.e., ground and non-ground). Thus, a ground mask $M_{g}$ can be obtained by checking the ground probability at the image coordinate (x,y) by $M_{g}\left(x,y\right)=sign\left(\Phi \left(I_{a}\left(x,y\right)\right)>0.5\right)$. Then, for a ground point $p_{xy}^{c}$ in camera coordinate system, we have

(12) $$\frac{n^{⊤}}{d}p_{xy}^{c}+1=0,$$

(13) $$K^{-1}D_{xy}p_{xy}=p_{xy}^{c},$$

where **n** is the ground unit normal vector in camera coordinate system and *d* is the distance from the camera to the ground. In the second equation, the homogeneous image point coordinate $p_{xy}$ is transformed into camera coordinate $p_{xy}^{c}$ by the intrinsic parameter matrix **K** and corresponding depth $D_{xy}$. The subscript indicates that the image coordinate of the point is (x,y). Thereafter, using the RANSAC based least square method in the ground area (mainly on the lower half image), we can estimate

(14) $$\frac{n}{d}=RANSAC\left(K,\left\{p_{xy},D_{xy}|M_{g}\left(x,y\right)=1,y>\frac{h}{2}\right\}\right)$$

with image height *h*.

After obtaining $\frac{n}{d}$, we calculate the average offset *o* of fitting points from the ground plane, as below:

(15) $$o=\frac{1}{|V_{r}|}\sum_{\left(x,y\right)\in V_{r}}\frac{|n^{⊤}p_{xy}^{c}+d|}{{\left||n|\right|}_{2}}$$

(16) $$s.t.\;V_{r}=\left\{(x,y)|R(x,y)=1\right\}.$$

In above equation, $V_{r}$ is the set of selected points in the RANSAC fitting phase. A point is only selected for fitting, when we have the inlier indicator R(i,j)=1. Points that are smaller than $\lambda_{r}\cdot o$ are selected as ground points to form the new label $l_{ground}$ ($p_{xy}^{c}$ and $p_{xy}$ are interchangeable by Equation (13)).

(17) $$l_{ground}=\left\{p_{xy}|\frac{|n^{⊤}p_{xy}^{c}+d|}{{\left||n|\right|}_{2}}<\lambda_{r}\cdot o\right\}.$$

With such a geometric prior, the new label is used in the cross entropy (CE) loss to re-learn the ground segmentation network. Thus, we have the ground self-learning loss.

(18) $$L_{r}=0.1\cdot e^{\lambda_{c}\cdot s}\cdot CE\left(\Phi \left(I_{a}\right),l_{ground}\right).$$

Due to the inaccuracy of the initial ground hypothesis, we slowly increase the weight of CE by the training step number *s*. In previous section, we are known that the original mutual information loss $L_{i}$ of the unsupervised method is learned through shallow semantics. Since the ground self-learning loss $L_{r}$ is learned through geometric information, combining those two losses can make their learning complementary to each other. Therefore, better ground segmentation results can be obtained (shown in the right of Figure 1).

#### 3.3.2. Plane Photometric Loss

Dynamic objects can cause problem for pose estimation. In turn, static objects are more valuable in estimating ego-poses. In the image, the ground can be considered as a huge static object and occupies a large image proportion. Thus, it has many good features, such as corner points and lane lines. In this approach, we propose a plane photometric loss which reconstructs the ground plane from one frame into another. For a certain plane point, the pixel coordinate transform between two frames $I_{a}$ and $I_{b}$ is given by

(19) $$p^{b}∼K^{-1}\left(R_{a}^{b}-\frac{t_{a}^{b}n^{⊤}}{d}\right)Kp^{a},$$

where $R_{a}^{b}$ and $t_{a}^{b}$ are inter-frame rotation and translation from $I_{a}$ to $I_{b}$, which are predicted by the pose network. *n* is the ground normal vector and *d* is the height of the camera. We have obtained $\frac{n}{d}$ using the RANSAC method. For more details, please see Appendix A.

Then, the plane photometric loss is defined as

(20) $$L_{H}=\frac{1}{\left|V_{H}\right|}\sum_{p\in V_{H}}\left({∥I_{a}\left(p\right)-I_{a^{\prime }}\left(p\right)∥}_{1}\cdot f\left(p\right)\right)$$

with f(p) as the probability of point *p* on the ground and obtained from the ground segmentation network. $V_{H}$ is the set of ground points which are successfully transformed between $I_{a}$ and $I_{b}$. Through this loss, the pose learning is strengthened through the static ground. Therefore, the ground segmentation results can help optimize the original SfM framework through the pose model. But this is not the case for dynamic objects, which leads to our third loss, the depth abnormal punishment loss.

#### 3.3.3. Depth Abnormal Punishment Loss

The self-supervised depth estimation method mainly relies on the position change between frames to estimate the depth information of the scene. For dynamic objects near the vanishing point in the image, their position change between frames is very small and thus can be mistaken for the same infinite as the vanish point. It is very common in the vehicle-following scene in the transportation task. However, since the ground normal direction has been obtained, it can be used to calculate out the ground horizon by

(21) $$K^{-⊤}\frac{n}{d}=\left[\begin{array}{c} a\\ b\\ c \end{array}\right],$$

(22) $$\frac{a}{c}x+\frac{b}{c}y+1=0.$$

Equation (22) represents the ground horizon. For details about the proof of this part, please see the Appendix B.

Assuming that $\left(x_{v},y_{v}\right)$ is the upper marginal center of the region of interest and $r_{w},r_{h}$ are the width and height, $\left(x_{v}-\frac{r_{w}}{2},y_{v}\right).\left(x_{v}+\frac{r_{w}}{2},y_{v}+r_{h}\right)$ are the corners in the diagonal direction of the region. In the early stage of training, since the normal vector is not stable, $\left(x_{v},y_{v}\right)$ is initialized by the image center point $\frac{w}{2}$,$\frac{h}{2}$ and w,h are the width and height of the image. At the end of training, $\left(x_{v},y_{v}\right)$ is chosen as the vanishing point in the same direction of the road and calculated by Equation (22). We obtain

(23) $$y_{v}=\left(1-\frac{ax_{v}}{c}\right)\frac{c}{b}.$$

Here we simply choose $x_{v}=\frac{w}{2}$, which is valid in our datasets. In the region of interest, the abnormal points produce relatively sharp increases in the depth, which is more obvious in the lateral direction of the image (e.g., comparing a vehicle with its nearby road surface). The set of abnormal points $V_{lv}$ can thus be found through the depth prediction as below:

(24) $$V_{lv}=\left\{\left(x,y\right)|D_{xy}>\frac{\lambda_{lv}}{r_{w}}\sum_{x^{\prime }=x_{v}-\frac{r_{w}}{2}}^{x_{v}+\frac{r_{w}}{2}}D_{x^{\prime }y}\right\}$$

and $\lambda_{lv}$ is a weight hyper-parameter. For such anomalies, they are removed from the original point set $V_{d}$ when calculating the photometric loss, expressed as

(25) $$L_{p}^{v}=\frac{1}{|V_{d}-V_{lv}|}\sum_{p\in V_{d}/V_{lv}}\left(\left(1-D_{diff}\right)\cdot L_{p}\left(p\right)\right).$$

Since $L_{p}$ was replaced by $L_{p}^{v}$, loss $L_{d}$ in Equation (6) is changed in the second stage, and this new loss is called $L_{d}^{\prime }$. At the same time, the abnormalities are punished in the loss

(26) $$L_{v}=\frac{1}{|V_{lv}|}\sum_{\left(x,y\right)\in V_{lv}}|D_{xy}-\frac{\lambda_{lv}}{r_{w}}\sum_{x^{\prime }=x_{v}-\frac{r_{w}}{2}}^{x_{v}+\frac{r_{w}}{2}}D_{x^{\prime }y}|.$$

By the understanding of the scene structure like vanishing point and the horizon lines, we can reduce the natural contradiction between the assumption of static objects between frames. In this way, the scene structure helps learning the SfM framework.

### 3.4. Entire Learning and Inference Framework

**Figure 5:**
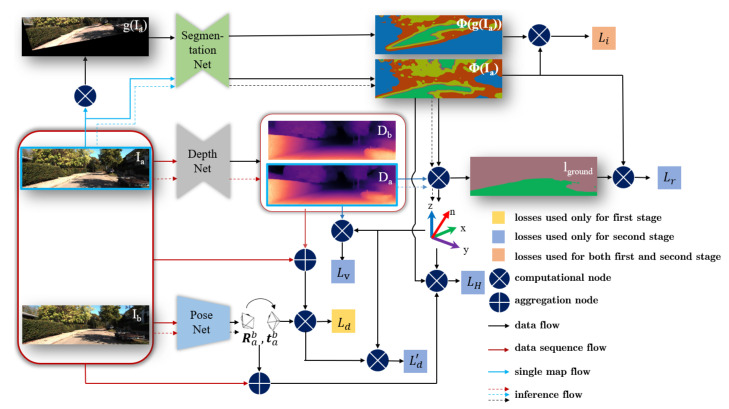
An overview of our entire learning and inference framework. Detailed description about the framework can be seen in the text.

Our entire learning and inference framework is shown in Figure 5. The loss $L_{i}$ and $L_{d}$ are used in the first learning stage (described in Section 3.1 and Section 3.2) while loss $L_{v}$, $L_{r}$ and $L_{H}$ are added in stage two. Additionally, the normal vector is used to improve the loss $L_{d}$, forming the modified loss $L_{d}^{\prime }$ (presented in Section 3.3). In the inference stage, as shown by the dotted line in Figure 5, the network predicts the scene depth, ego-motion, and ground segmentation. Based on the results of ground segmentation and depth estimation, the ground normal vector is calculated using Equation (14). Through these methods, a powerful scene structure estimator is constructed, and its sub-tasks promote each other through our proposed losses.

## 4. Experiment

### 4.1. Dataset

In the research field of autonomous driving, the KITTI data set (http://www.cvlibs.net/datasets/kitti/index.php) is currently one of the largest evaluation datasets for computer vision algorithms such as stereo imaging, optical flow, visual odometry, 3D object detection and tracking, depth and pose estimation by vehicle-mounted cameras and lidar. In our experiments, we use the KITTI raw data and KITTI odometory data. The KITTI raw data is composed of 78 short sequences of about 45,000 images. We follow the protocol of Eigen’s Split [20] same as in works [9,10,11,12,13,27,38,39] and divide these 78 sequences into two subsets: 697 images for testing, and the rest are for training and validation. The Cityscapes (https://www.cityscapes-dataset.com) is a large-scale benchmark for segmentation tasks of traffic scene. It is composed of 5488 short sequences in 50 cities, about 70,000 images, of which 5000 are finely marked and 20,000 are roughly marked for segmentation task. Although Cityscapes is unsuitable for evaluating the estimation performance of depth, pose and ground normal vector, it contains video streams. Thus, it can be used in the unsupervised pre-training process like in works [12,13,27]. Moreover, the Cityscapes dataset provides annotations for the ground segmentation which is not available in the KITTI dataset. Hence, we use Cityscapes (i.e., the official validation set of 500 marked images) for evaluating our ground segmentation subnetwork. Since we only focus on the binary ground segmentation task, we also make adaption of the official evaluation tool in our experiments.

### 4.2. Implementation Details

#### 4.2.1. Training Configuration

The hyper-parameters used in the training are as follows. In the first stage, for the coefficients in loss $L_{d}$ (Equation (6)), we follow the work [27,31], so that the experimental comparison with them should be fair. Thus, coefficients α, β, γ are respectively set to 1.0, 0.1, 0.5, and $\lambda_{s}=0.85$. In the second stage, the existing hyper-parameters in the first stage remain the same, and we set $\lambda_{r}=0.3$, $\lambda_{c}=\frac{\ln10}{50000}$, $\lambda_{lv}=2$, which are searched through experiments on the validation set. Regarding the ROI selection, according to the statistics of used images, we found that an area near the vanishing point with a size of $\frac{w}{3}\times \frac{h}{4}$ can well handle the abnormal depth values (*h* and *w* are respectively the height and width of the image). Because we use a unified image size of 832×256, we set $r_{w}=277$ and $r_{h}=64$. In the training process, we randomly select 1000 batches as an epoch. In the first training stage we train 200,000 iterations which is the same in Bian et al. [27] while in the second training stage we train 80k iterations. This configuration is based on experiments on the validation set. In the second phase, since the ground segmentation is not accurate enough in the early period of training, the location of the vanishing point calculated by the normal vector fluctuates too much, which will damage the depth estimation. Hence, the upper marginal center of ROI is approximated by the image center in the first 50,000, but in the later period when the road surface estimation becomes gradually stable and accurate, the calculation of vanishing point also becomes more accurate. Therefore, in the last 30,000, the vanishing point is calculated by the ground normal vector. The learning rate is set to $10^{-4}$ in the first stage and reduces to $5\times 10^{-5}$ in the second stage in order to make the learning more stable. The batch size is set to 4.

#### 4.2.2. Network Structure

For the depth and pose estimation network, we adopt the DispRes-Net [27], and PoseNet [27] as backbone. These backbone structures are the same as in works [13,27], so that the subsequent experimental comparison should be fair. The ground segmentation network has the same structure as the depth network, with the output header divided into 5 categories due to the reason that more categories can be conducive to mutual information learning, as pointed out in [31]. Here one category is for the ground, and the other four together form the non-ground category.

#### 4.2.3. Environment

The environment for training and testing our approach is a desktop with Intel(R) Xeon(R) CPUs E5-2667 v3 of 3.20 GHz by Intel and a memory of 64G DDR4 (Samsung, Seoul, South Korea). The GPU we use is GTX 1080 Ti (Nvidia, Santa Clara, California, United States). The network is implemented by PyTorch-1.2.0. The version of python is 3.6.8 and the operating system is Ubuntu 16.04.6 LTS.

### 4.3. Experimental Results

This part mainly carries out comparison experiments from four aspects, namely the depth estimation, pose estimation, ground normal vector quantitative measurement, and unsupervised ground estimation.

#### 4.3.1. Depth Estimation Results

For testing the depth estimation network, the KITTI raw data is divided into training and test set according to the Eigen’s Split [20] same as in related works [9,11,12,13]. Additionally, we also test the performance of the network with pre-training on the Cityscapes data.

**Figure 6:**
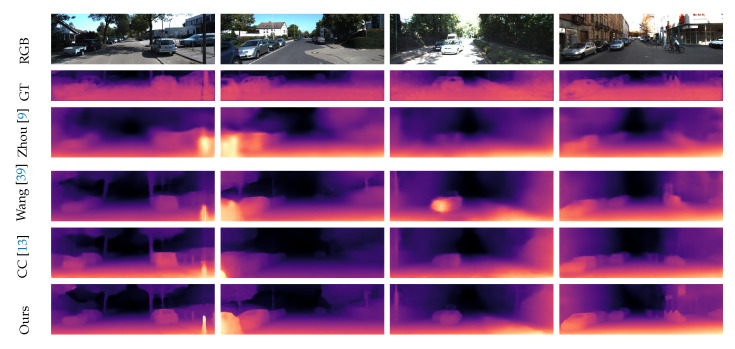
Qualitative results of scene depth estimation on KITTI raw dataset. Compared to other methods, our method has clearer outline and more details in estimated depth map. This can be credited to the use of geometric information in our method.

The test results are given in Table 1. For the depth evaluation, the error metrics used are the absolute relative error (AbsRel), the square relative error (SqRel), the root mean square error (RMS), and the root mean square logarithmic error (RMSlog). For the accuracy, three thresholds are used for evaluation. Above metrics are widely used in depth estimation tasks to comprehensively consider errors and accuracy. For calculation details about these metrics, please refer to the Appendix A, Appendix B, Appendix C and Appendix D. The compared methods are mainly from three groups: using point cloud as groundtruth (GT) to directly perform supervised (S) learning, using binocular camera calibration information for semi-supervised (SS) learning, and using video streams for unsupervised (US) learning. In comparison with other methods, it can be seen that whether our approach is only trained on the KITTI or on Cityscapes + KITTI, it achieves significant improvement on depth estimation in terms of unsupervised approaches. Compared with the baseline model SC-SfMlearner [27], we outperform it in all depth-related error and accuracy metrics. This is due to the employment of our depth abnormal punishment loss, which uses the structural information of the scene, i.e., the normal vector, to suppress the abnormal depth. Compared with the semi-supervised methods, except for a slight lag in the absolute relative error (AbsRel), our approach (trained on CS + K) surpasses the best method [25] on other metrics. In comparison with supervised depth estimation methods, our approach achieves the second place, only with a minor gap to the top method [22]. For a more intuitive impression, we select some test examples as qualitative results shown in Figure 6. However, due to the fact that the lidar cloud is very sparse while the evaluated method generates a dense depth map, a direct qualitative comparison will not be intuitive. Therefore, we resort to the depth completion method by DeepLidar [40]. This method uses an encoder-decoder structure to effectively fuse the dense color image and sparse lidar points. It also uses a network to estimate surface normals as the intermediate representation for dense depth map completion. It is one of the top performed methods in the KITTI depth completion benchmark for lidar data. Due to the lack of point cloud data in the upper part of the image, the corresponding completion cannot be performed. Thus, we truncate this part. Among the compared methods, Zhou et al. [9] were the first to use monocular image for unsupervised depth estimation. In order to solve the problem of dynamic objects, they use explainability to mask dynamic objects. Wang et al. [39] use a differentiable implementation for direct visual odometry, along with a depth normalization strategy. The CC [13] method uses four sub-networks and uses a training method similar to the EM algorithm. Compared to these methods, the clearer outline and details in estimated depth map are more clear, this is due to the geometric information such as ground surface and scene structure used in our network learning and demonstrate the effectiveness of our approach.

#### 4.3.2. Ego-Pose Estimation Results

**Figure 7:**
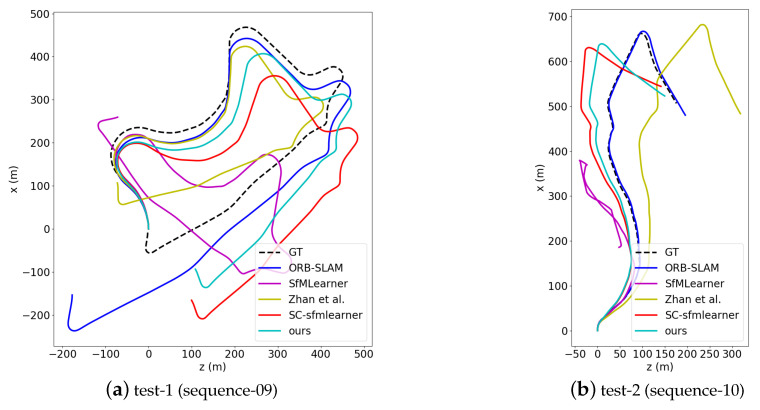
Qualitative results on the KITTI odometry test set. (**a**) is the result on test-1, (**b**) is the result on test-2. In comparison with unsupervised methods, our estimated trajectory is relatively closer to the GT in both sequences.

In this part we conduct the experiment on the KITTI odometry dataset [18]. The model is trained and verified on the sequence 00–08 and tested on sequence 09–10. For clarity, the sequence 09 and 10 are respectively renamed as test-1 and test-2 in Figure 7 and Table 2. The experimental results are shown in Table 2. The Oriented FAST and Rotated BRIEF-Simultaneous Localization and Mapping (ORB-SLAM) [43] system (without loop closure) is reported as a reference. It can be seen that our method achieves a certain improvement on the pose estimation in terms of unsupervised methods, especially compared with baseline model SC-SfMleaner [27]. This is because our plane photometric loss improves the weight of static objects in the optimization process. Thanks to the static ground features, the pose model is better optimized, which shows that the understanding of the scene geometry does help the optimization of the model. However, compared with the traditional geometry based method, i.e., the ORB-SLAM, there is still a gap for deep learning based methods in pose estimation. A qualitative comparison result is given in Figure 7, from which it can also be seen that compared to other unsupervised methods, our estimated trajectory is relatively closer to the GT. Especially compared with the baseline model SC-SfMleaner [27], in both sequence test-1 and test-2, our accuracy has been significantly improved. This is also consistent with the results in Table 2.

#### 4.3.3. Ground Normal Vector Estimation Results

**Figure 8:**
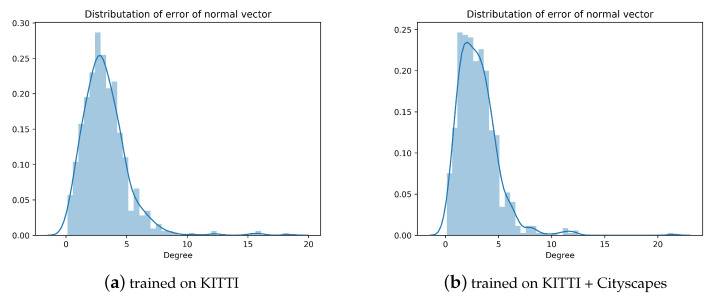
The error distribution of predicted ground normal vector. (**a**) shows prediction errors of the model trained on the KITTI raw data, while in (**b**) the corresponding model is trained on both Cityscapes and KITTI raw data.

**Figure 9:**
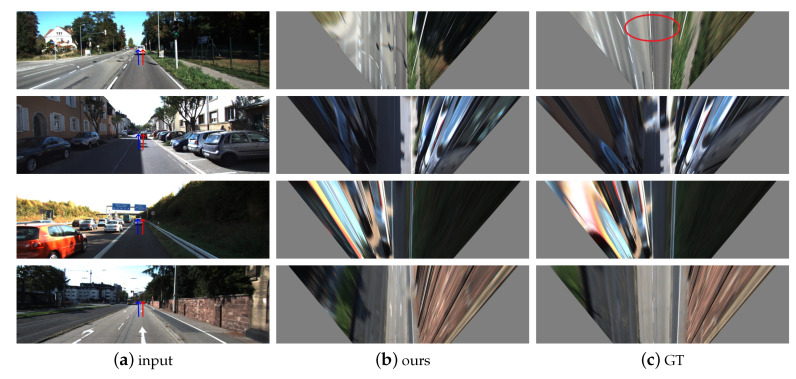
(**a**) is the original image. The red arrow denotes our predicted ground normal vector, and the blue one is the groundtruth calculated using extrinsic parameters. (**b**) is the BEV image transformed using our predicted normal vector while (**c**) is the BEV transformed using the groundtruth normal vector. The parallelism of the lane lines near the red circle is relatively low, indicating that the groundtruth value is sometimes not accurate enough.

In this experiment, we use exactly the same dataset as for the depth estimation model. There is only a few works to estimate ground normal vectors from monocular video. We compare the existing works with our approach and report the result in Table 3. We surpass the hidden markov model (HMM) method [44] by about $0.9^{\circ }$. With training on Cityscapes+KITTI, the error is further reduced by $0.2^{\circ }$. However, there is still a gap between our method and the supervised GroundNet [15]. The GroundNet requires labels for the ground, depth and normal vector, while we only rely on continuous video streams. We also give the error distribution of our estimated normal vector on the test set in Figure 8a,b. It can be seen that errors are mainly concentrated around $3^{\circ }$. In order to perceive the estimation result of normal vector more intuitively, we project the normal vector into the original image, as shown in Figure 9a. Since the error is small, it is difficult to detect the change especially in the pitch angle, which is yet important in the field of automated driving. Therefore, we transform the image to a bird’s eye view (BEV). Through the parallelism of the lane lines, we can intuitively judge the quality of the pitch angle. The result is shown in Figure 9. It can be seen that the deviation of parallelism by our approach is relatively small. Note that the reference value (Figure 9b) directly calculated by the given extrinsic parameters is sometimes inaccurate, which implies that our actual error is likely to be smaller than what we reported in Table 3. In the absence of labeled data, our normal vector estimation has achieved relatively good results.

#### 4.3.4. Unsupervised Ground Segmentation Results

Due to the lack of pixel-level annotations on KITTI data, this part of experiment is conducted on the Cityscapes. Here we take all points belonging to the ground plane as positive samples and test our approach on the official validation set.

To demonstrate the effectiveness of our joint learning, we also extract the ground segmentation network from our architecture and perform supervised training on Cityscapes. The results are given in Table 4. It can be seen that even compared with the supervised method, we are still ahead of 9% in terms of the IOU accuracy. This is because our mutual information loss allows the network to learn better feature representations and improve the generalization ability of the network. For the simple supervised method, the representation learning is not sufficient, so the result is not as good as unsupervised methods with geometric correction. This also proves that the learning of semantic information can be promoted by adding geometric prior.

### 4.4. Ablation Experiment

Here we give a deep exploration on the effectiveness of those three losses used in the second training phase by ablation studies. Since the plane photometric loss (PPL) needs the ground segmentation result, which strongly depends on the ground self-learning loss (GSFL), we combine both losses together as GSFL + PPL. The depth abnormal punishment loss here is abbreviated as APL. We compare the performance of GSFL + PPL and APL, which are separately integrated with our approach. The depth estimation result is shown in Table 5, the improvement of APL is very obvious, while the effect of GSFL + PPL is relatively weak. The pose estimation result is shown in Table 6, we can notice that GSFL + APL does improve the estimation of the ego-motion, while the improvement by APL is not so obvious. This is consistent with our expectation. We expect that GSFL + PPL can be used to enhance the optimization of static objects in ego-motion estimation, and the abnormal depth can be improved through APL. The result of ground segmentation is given in Table 7 and shows that the effect of GSFL is very obvious with the IOU accuracy increased by 35%. This fully illustrates the importance of geometric prior information. We can observe this improvement qualitatively in the right part of Figure 1, in which the shadow area is also detected by adding geometric prior constraints through GSFL.

## 5. Conclusions

Estimation of scene structures (including scene depth, ego-pose, ground normal vector and ground segmentation) by a camera sensor is a crucial task for automated driving and robotics. In supervised methods, learning scene structures often requires groundtruth labels obtained by expensive sensors such as lidar, which limits their application. In existing unsupervised methods, estimation of the scene depth can also be affected by dynamic objects which do not meet the static assumption. In order to alleviate these problems, we propose a completely unsupervised learning framework for scene structure estimation by monocular camera. In this approach, we innovatively proposed three losses in the joint learning process. Through these three losses, the ground segmentation results can be corrected by the estimated geometric prior while the depth estimation is improved by reducing the error of transformed ground plane. And the impact of dynamic objects can be suppressed using the estimated ground normal vector. Experimental results on the KITTI and Cityscape datasets demonstrate that the depth and pose estimation results have been significantly improved by our approach. We also achieve better ground segmentation and normal vector estimation results in terms of unsupervised learning methods. In the future, we will continue to explore how to combine more scene structure priors to further improve the framework.

## Figures and Tables

**Table 1:** The result of depth estimation. K represents the use of KITTI raw data for training while CS + K represents the use of both the Cityscapes and the KITTI raw data. B represents the training with binocular images. D represents using depth groundtruth as supervision signal, and M is for monocular video. S represents the supervised method, SS represents semi-supervised method and US represents unsupervised method. For the error, the lower is better and for the accuracy, the higher is better. The details of the indicators can be seen in Appendix D.

Learning	Method	Datasets	Error ↓				Accuracy ↑		
			AbsRel	SqRel	RMS	RMSlog	<1.25	${<1.25}^{2}$	${<1.25}^{3}$
S	Eigen et al. [20]	K (D)	0.203	1.548	6.307	0.282	0.702	0.890	0.958
	Liu et al. [41]	K (D)	0.202	1.161	6.523	0.275	0.678	0.895	0.965
	Kuznietsov et al. [22]	K (B + D)	**0.113**	**0.741**	**4.621**	**0.189**	**0.862**	**0.960**	**0.986**
SS	Garg et al. [42]	K (B)	0.152	1.226	5.849	0.246	0.784	0.921	0.967
	Zhan et al. [26]	K (B)	0.144	1.391	5.869	0.241	0.803	0.928	0.969
	Godard et al. [25]	K (B)	0.148	1.344	5.927	0.247	0.803	0.922	0.964
	Godard et al [25]	CS + K (B)	**0.124**	**1.076**	**5.311**	**0.219**	**0.847**	**0.942**	**0.973**
US	Zhou et al. [9]	K (M)	0.208	1.768	6.856	0.283	0.678	0.885	0.957
	Yang et al. [38]	K (M)	0.182	1.481	6.501	0.267	0.725	0.906	0.963
	Mahjourian et al. [10]	K (M)	0.163	1.240	6.220	0.250	0.762	0.916	0.968
	Wang et al. [39]	K (M)	0.151	1.257	5.583	0.228	0.810	0.936	0.974
	Geonet-VGG [11]	K (M)	0.164	1.303	6.090	0.247	0.765	0.919	0.968
	Geonet-Resnet [11]	K (M)	0.155	1.296	5.857	0.233	0.793	0.931	0.973
	DF-Net [12]	K (M)	0.150	1.124	5.507	0.223	0.806	0.933	0.973
	CC [13]	K (M)	0.140	1.070	5.326	0.217	0.826	0.941	0.975
	SC-SfMLearner [27]	K (M)	0.137	1.089	5.439	0.217	0.830	0.942	0.975
	ours	K (M)	**0.135**	**1.006**	**5.336**	**0.212**	**0.833**	**0.944**	**0.977**
US	Zhou et al. [9]	CS + K (M)	0.198	1.836	6.565	0.275	0.718	0.901	0.960
	Yang et al. [38]	CS + K (M)	0.165	1.360	6.641	0.248	0.750	0.914	0.969
	Mahjourian et al. [10]	CS + K (M)	0.159	1.231	5.912	0.243	0.784	0.923	0.970
	Wang et al. [39]	CS + K (M)	0.148	1.187	5.496	0.226	0.812	0.938	0.975
	Geonet-Resnet [11]	CS + K (M)	0.153	1.328	5.737	0.232	0.802	0.934	0.972
	DF-Net [12]	CS + K (M)	0.146	1.182	5.215	0.213	0.818	0.943	0.978
	CC [13]	CS + K (M)	0.139	1.032	5.199	0.213	0.827	0.943	0.977
	SC-SfMLearner [27]	CS + K (M)	0.128	1.047	5.234	0.208	0.846	0.947	0.976
	ours	CS + K (M)	**0.126**	**0.943**	**5.084**	**0.203**	**0.849**	**0.949**	**0.978**

**Table 2:** The estimation results on KITTI odometry dataset. $t_{err}$ is average translational drift error. $r_{err}$ is average rotational drift error. The ORB-SLAM is a traditional visual SLAM method and is used as a reference. The best results for methods based on unsupervised learning is highlighted.

Methods	Test-1 (Sequence 09)		Test-2 (Sequence 10)	
	$t_{\mathrm{err}}\left(\%\right)$	$r_{\mathrm{err}}\left({}^{\circ }/100m\right)$	$t_{\mathrm{err}}\left(\%\right)$	$r_{\mathrm{err}}\left({}^{\circ }/100m\right)$
ORB-SLAM [43]	15.30	0.26	3.68	0.48
Zhou et al. [9]	17.84	6.78	37.91	17.78
Zhan et al. [26]	11.93	3.91	12.45	**3.46**
SC-SfMlearner [27]	11.2	3.35	**10.1**	4.96
ours	**9.36**	**2.61**	10.25	3.84

**Table 3:** Comparison of normal vector estimation. The groundtruth value is calculated using extrinsic parameters. K means only using KITTI raw data for training, and CS + K means using Cityscapes and KITTI raw data.

Methods	Error/Deg
GroundNet [15] (Supervised)	**0.70**
HMM [44] (Unsupervised)	4.10
ours (K) (Unsupervised)	3.23
ours (CS + K) (Unsupervised)	**3.02**

**Table 4:** Test results for ground segmentation using our network structure with or without supervision.

Methods	IOU
Supervision	0.74
Unsupervised	**0.83**

**Table 5:** The depth ablation experiments are carried out on the KITTI raw data. GSF, PPL, and APL respectively represent the ground self-learning loss, the plane photometric loss and the depth abnormal punishment loss.

Methods	Datasets	Error ↓				Accuracy ↑		
		AbsRel	SqRel	RMS	RMSlog	<1.25	${<1.25}^{2}$	${<1.25}^{3}$
Basic	K	0.137	1.091	5.441	0.217	0.830	0.942	0.975
Basic + GSFL + PPL	K	0.136	1.103	5.417	0.215	**0.835**	0.944	0.976
Basic + GSFL + PPL + APL	K	**0.135**	**1.006**	**5.336**	**0.212**	0.833	**0.944**	**0.977**

**Table 6:** The ego-motion estimation ablation experiments are carried out on the KITTI odometry dataset. GSF, PPL, and APL respectively represent the ground self-learning loss, the plane photometric loss and the depth abnormal punishment loss.

Methods	Test-01 (Sequence 09)		Test-02 (Sequence 10)	
	$t_{\mathrm{err}}\left(\%\right)$	$r_{\mathrm{err}}\left({}^{\circ }/100m\right)$	$t_{\mathrm{err}}\left(\%\right)$	$r_{\mathrm{err}}\left({}^{\circ }/100m\right)$
Basic(K)	11.24	3.34	10.07	4.91
Basic + GSFL + PPL(K)	**9.34**	2.63	**9.51**	3.97
Basic + GSFL + PPL + APL(K)	9.36	**2.61**	10.14	**3.84**

**Table 7:** Ground segmentation results with or without the GSFL loss.

Methods	IOU
Basic	0.48
Basic + (GSFL)	**0.83**

## Appendix A. Homography Transformation

Given two consecutive image frames, a point $p^{c}$ on the first frame within the camera coordinate system applies the camera imaging model

(A1) $$p_{1}=Kp_{1}^{c}$$

with intrinsic matrix **K**. Here homogeneous image coordinates $p_{1}$ are used. The motion between the first frame and the second frame is represented by [R, t], which denote the rotation and translation, respectively. Then, the point $p_{1}^{c}$ is transformed into the camera coordinate system of the second frame, satisfying following equation

(A2) $$p_{2}=K\left(Rp_{1}^{c}+t\right).$$

Considering point $p_{1}^{c}$ on the ground plane, it satisfies equation

(A3) $$n^{⊤}p_{1}^{c}+d=0$$

with the camera height *d* and ground normal vector **n**. The above equation can be further modified as

(A4) $$-\frac{n^{⊤}p_{1}^{c}}{d}=1,$$

(A5) $$t=-\frac{{tn}^{⊤}p_{1}^{c}}{d}.$$

Introducing above equation into Equation (A2), we obtain

(A6) $$p_{2}=K\left(Rp_{1}^{c}-\frac{tn^{⊤}p_{1}^{c}}{d}\right),$$

(A7) $$p_{2}=K\left(R-\frac{tn^{⊤}}{d}\right)p_{1}^{c}.$$

Introducing Equation (A1) into above equation, we can get

(A8) $$p_{2}=K\left(R-\frac{tn^{⊤}}{d}\right)K^{-1}p_{1},$$

which is the formula for transforming planes between frames.

## Appendix B. Calculation of the Horizon

Considering the set *V* of infinity points on the ground in the camera coordinate system, a point $q^{c}\in V$ satisfies the camera imaging model

(A9) $$p={Kq}^{c}$$

with homogeneous image coordinate **p**, which indicates the infinity point on the ground plane. Since the line between $q^{c}$ and the camera origin is perpendicular to **n**, we have

(A10) $$q^{c⊤}n=0.$$

Introducing Equation (A9) into above equation, we have

(A11) $$p^{⊤}K^{-⊤}n=0.$$

In this paper, there is an additional coefficient on the left side of Equation (A11), but it does not affect the result, because the right side is 0. Since **p** is the coordinate of the infinity point on the ground, all such points constitute the horizon in the image. Thus, the horizon equation is

(A12) $$K^{-⊤}n=\left[\begin{array}{c} a\\ b\\ c \end{array}\right],$$

where the right side of above equation denotes the parameters of the horizon line.

Thus, the horizon equation can be formulated with image point **p** by

(A13) $$p=\left[\begin{array}{c} x\\ y\\ 1 \end{array}\right],$$

(A14) $$\frac{a}{c}x+\frac{b}{c}y+1=0.$$

## Appendix C. Calculation of the Loss Based on SSIM

Given two small patches **x** and **y**, the SSIM mainly considers three aspects, namely the luminance comparison l(x,y), contrast comparison c(x,y), and structure comparison s(x,y). The similarity for two patches is represented as

(A15) S(x,y)=l(x,y)·c(x,y)·s(x,y).

The luminance comparison is defined as follows

(A16) $$l\left(x,y\right)=\frac{2\mu_{x}\mu_{y}+C_{1}}{\mu_{x}^{2}+\mu_{y}^{2}+C_{1}},$$

where $\mu_{x},\mu_{y}$ respectively denote the mean of pixel value for small patch **x** and **y**, which are of 5×5 in our work. And $C_{1}$ is a constant and set to 0.02 as in the work [37].

The contrast comparison is defined as

(A17) $$c\left(x,y\right)=\frac{2\sigma_{x}\sigma_{y}+C_{2}}{\sigma_{x}^{2}+\sigma_{y}^{2}+C_{2}},$$

where $\sigma_{x},\sigma_{y}$ respectively denote the standard deviation of pixel values in patch **x** and **y**. The constant $C_{2}$ is set to 0.06, same as in the work [37].

The structural comparison is defined as

(A18) $$s\left(x,y\right)=\frac{\sigma_{xy}+C_{3}}{\sigma_{x}\sigma_{y}+C_{3}}$$

and the $\sigma_{xy}$ is

(A19) $$\sigma_{xy}=\frac{1}{N-1}\sum_{i=1}^{N}\left(x_{i}-\mu_{x}\right)\left(y_{i}-\mu_{y}\right).$$

$C_{3}$ is set to $\frac{C_{2}}{2}$. *N* denotes the pixel number of the patch. Since the photometric value subtracts the mean and is divided by the standard deviation, the structural comparison excludes the effects of luminance and contrast.

In summary, the SSIM for two patches can be expressed as

(A20) $$\mathrm{SSIM}\left(x_{j},y_{j}\right)=\frac{\left(2\mu_{x_{j}}\mu_{y_{j}}+C_{1}\right)\left(\sigma_{x_{j}y_{j}}+C_{2}\right)}{\left(\mu_{x_{j}}^{2}+\mu_{y_{j}}^{2}+C_{1}\right)\left(\sigma_{x_{j}}^{2}+\sigma_{y_{j}}^{2}+C_{2}\right)}.$$

So the loss based on SSIM in the paper can be expressed as

(A21) $${\mathrm{SSIM}}_{aa^{\prime }}\left(p\right)=\mathrm{SSIM}\left(I_{a}\left(p\right),I_{a^{\prime }}\left(p\right)\right).$$

The $I_{a}\left(p\right),I_{a^{\prime }}\left(p\right)$ here specifically refer to the patches centered on pixel *p*. Based on the above description, SSIM considers more comprehensive information than other simple image difference measurements. It is more in line with the requirements in our method.

## Appendix D. The Metrics Used for Depth Estimation

For depth estimation, we denote $D_{i}^{*}$ as the ground truth and $D_{i}$ as the estimated value. *N* is the number of involved points. The absolute relative error is defined as

(A22) $$\mathrm{AbsRel}=\frac{1}{N}\sum_{i=1}^{N}\frac{\left|D_{i}-D_{i}^{*}\right|}{D_{i}^{*}}.$$

The square relative error is

(A23) $$SqRel=\frac{1}{N}\sum_{i=1}^{N}\frac{{\left|D_{i}-D_{i}^{*}\right|}^{2}}{D_{i}^{*}}.$$

The root mean square error is

(A24) $$RMS=\sqrt{\frac{1}{N}\sum_{i=1}^{N}{\left|D_{i}-D_{i}^{*}\right|}^{2}}.$$

The root mean square logarithmic error is

(A25) $$\log RMS=\sqrt{\frac{1}{N}\sum_{i=1}^{N}{\left|\mathrm{lg}D_{i}-\mathrm{lg}D_{i}^{*}\right|}^{2}}.$$

The accuracy is defined as

(A26) $$\frac{1}{N}\sum_{i}^{N}\mathrm{sign}\left(\max\left(\frac{D_{i}}{D_{i}^{*}},\frac{D_{i}^{*}}{D_{i}}\right)<T\right).$$

with threshold *T*. Three different thresholds ($1.25,1.25^{2},1.25^{3}$) are used in the accuracy metric as in works [9,10,11,12,13,27,38,39].
